# Development and Characterization of a Peptide-Bisphosphonate Nanoparticle for the Treatment of Breast Cancer

**DOI:** 10.1021/acs.molpharmaceut.4c00299

**Published:** 2024-08-28

**Authors:** Kimberley Glass, Cory Fines, Paula Coulter, Lynn Jena, Helen O. McCarthy, Niamh Buckley

**Affiliations:** School of Pharmacy, Queen’s University Belfast, 97 Lisburn Road, BT9 7BL Northern Ireland, U.K.

**Keywords:** bisphosphates, breast cancer, RALA, nanomedicine, risedronate

## Abstract

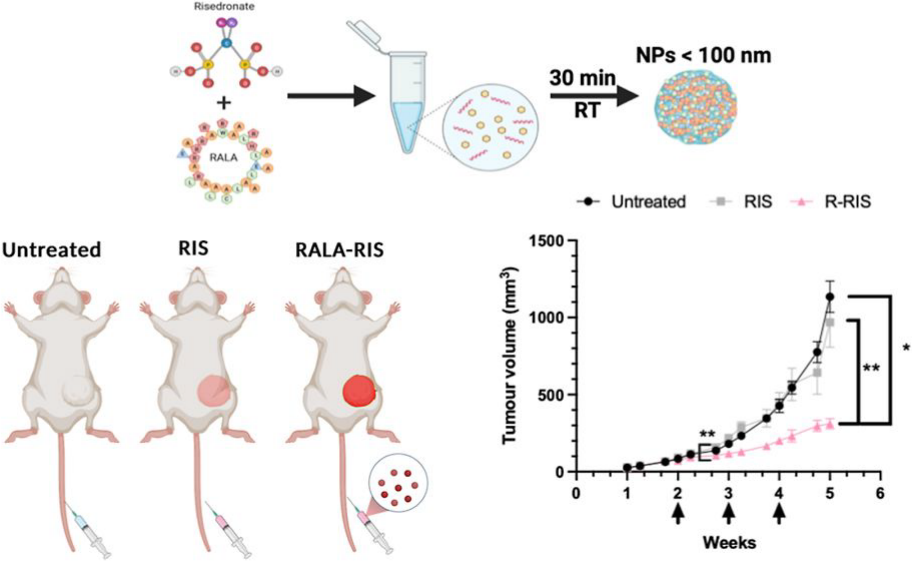

In women, breast cancer (BC) is the most common cancer, and despite advancements in diagnosis and treatment, 20–30% of early stage BC patients develop metastatic disease. Metastatic BC is deemed an incurable disease, which accounts for 90% of BC related deaths, with only 26% of metastatic patients reaching a 5 year survival rate. Therefore, there is an unmet need for the prevention or treatment of metastasis in early stage breast cancer patients. Bisphosphonates (BPs) are potent inhibitors of bone resorption and are extensively used for the prevention of osteoporosis and other skeletal disorders, as well as for the treatment of secondary bone cancer in BC patients. Furthermore, the direct anticancer activity of BPs has been established in primary tumor models. However, these studies were limited by the need for dosages far above the clinical range to overcome BPs’ high affinity for bones and poor accumulation in the tumor itself, which leads to toxicity, including osteonecrosis of the jaw. To decrease BP dosage, increase bioavailability, and direct anticancer activity, we used the RALA (R-) peptide delivery system to form highly stable NPs with the nitrogen containing BP, risedronate (R-RIS). In vitro studies showed that, in comparison to RIS, R-RIS nanoparticles increased cytotoxicity and reduced metastatic features such as proliferation, migration, invasion, and adhesion of metastatic BC cells to bones. Furthermore, in an in vivo model, R-RIS had increased tumor accumulation while still maintaining similar bone accumulation to RIS alone. This increase in tumor accumulation corresponded with decreased tumor volume and lungs metastasis. R-RIS has great potential to be used in combination with standard of care chemotherapy for the treatment of primary BC and its metastasis while still having its bone resorption inhibiting properties.

## Introduction

Between 20 and 30% of patients diagnosed with breast cancer (BC) will eventually develop deadly metastasis, which can occur even decades after primary diagnosis and surgical resection of the primary tumor.^2^ Bone is the most common site of metastasis in BC patients, with up to 65–75% of stage IV BC patients developing skeletal metastases.^3^ Metastatic disease is the limiting factor in long-term patient survival, and the development of effective treatments to prevent or treat metastasis is among the most outstanding challenge in current experimental and clinical cancer research.^4^

Nitrogen-containing bisphosphonates (N-BPs) are potent inhibitors of osteoclast activity and are recommended as adjuvant therapy for postmenopausal BC patients at high risk of treatment induced-bone loss, metastasis, and for other skeletal related events.^1^ N-BPs such as risedronate (RIS) and zoledronate (ZOL), act on the mevalonate pathway by inhibiting the key enzyme farnesyl diphosphate synthase (FPP synthase).^5^ This thereby depletes the cell of the isoprenoid lipids FPP and geranylgeranyl pyrophosphate (GGPP),^6^ which are needed for the post translational prenylation of members of the small G-protein superfamily, including small GTPases such as Rac, Rho, and Ras.^7^ Several clinical trials have shown adjuvant N-BP in early stage breast cancer (ESBC) patients improves survival and reduces skeletal/extra-skeletal metastasis.^8^ Specifically, after successful in vivo studies showing RIS was effective in reducing metastasis,^9−11^ clinical trials showed RIS was effective in reducing bone loss in ESBC as well as patients taking aromatase inhibitors.^12−15^ However, the benefits of N-BPs are limited to postmenopausal or induced menopause patients, as there is less bone-turnover in premenopausal women which is needed for the release and uptake of N-BPs.

Of further interest, besides N-BPs role on the metastatic niche and bone TME, in vitro studies have shown N-BPs can also directly act on cancer cells, decreasing cancer proliferation, viability, migration, and cell adhesion.^16−21^ However, outside of the bone, N-BPs have poor bioavailability and pharmacokinetics, limiting its direct antitumor efficacy. When given orally, only 0.6–1% of BP administration reaches systemic circulation and, even when given intravenously, 70% of the BPs bind to skeletal tissue with the remainder excreted unmetabolised through the kidneys.^22−24^ To mimic the clinical situation, it could be argued that the three to five times higher bone turnover in mice would require an equivalently higher N-BP dosage.^25^ However, most animal studies even exceed this high dose, reaching up to 40 times the dosing regimens currently used in the clinic.^26−28^ This is not translatable, as too high of dosages or prolonged treatment with BPs can cause side effects including fever, increased bone pain and osteonecrosis of the jaw (ONJ).^29^ Therefore, in hope of increasing direct antitumor efficacy and possibly expanding patient efficacy to premenopausal women, there is a need for an improved delivery system for N-BPs to increase bioavailability and pharmacokinetics.

To tackle this need, we have introduced a novel cell-penetrating peptide, RALA, as an attractive delivery platform of N-BPs for the treatment of early stage and advanced BC.^30^ RALA has successfully been used in vitro and in vivo to deliver a wide range of cargo including plasmid DNA, mRNA and siRNAs to form stable NPs that exert antitumor effects in a range of cancer types with no observed toxicity following repeated injections.^31−33^ The most attractive characteristic of using RALA as a delivery system is the simplicity involved in the formulation process. RALA is a positively charged particle that self-assembles with negatively charged cargo.^34^ Unlike many other drug delivery platforms, this method requires only three components and a small time frame of self-assembly that is scalable and readily compatible with commercial automated microfluidic systems for large scale manufacture.^34^ Past studies have shown BPs are effective in reducing metastasis to the bone and other organs in BC in vivo models; however, none demonstrated any direct antitumor efficacy. In our own previous studies, we demonstrated RALA complexed N-BPs has an in vitro cytotoxic efficacy in glioma and prostate cancer cell lines and even MDA-MB-231 BC cells, however BC in vivo models were not explored.^31,35^ Therefore, there was a gap in knowledge of whether RALA complexed N-BPs could be used to treat the primary breast tumor in a mouse model. Due to the prevalence of BPs already in BC treatment and their currently poor accumulation in the primary tumor, we aimed to optimize their delivery using RALA nanoparticles in hopes of utilizing BPs in primary BC treatment. ZOL is the most potent N-BP, which is given in the adjuvant setting to postmenopausal women with BC via IV infusion over 15 min every 42 days for 3 cycles.^36^ However, this dosing schedule could not be replicated in a mouse model due to accelerated tumor growth rates. Furthermore, the AZURE trial was the first study to explore the antitumor activity of ZOL with adjuvant chemotherapy. A total of 3360 patients with stage II/III early BC were randomized to receive either standard of care chemotherapy with or without ZOL (4 mg) for 5 years. There was no difference between groups in disease free survival (DFS), overall survival (OS) and distant recurrences however ZOL did decrease bone metastasis.^37^ ZOL had no effect on early BC, and since bone metastasis were not the focus of this study but direct antitumor effects, we focused our attention to another clinically approved N-BP, RIS. While we delivered RIS intravenously in our in vivo studies, we were able to deliver RIS every week as it is typically given orally once weekly in BC patients.^38^ We were successful in developing a nanoparticle formulation of RALA-risedronate (R-RIS) that increased the cytotoxicity of risedronate on cancer cells and decreased their metastatic potential in vitro. We then demonstrated the ability of R-RIS to decrease primary tumor growth in an in vivo model at a more clinically translatable dosage.

## Materials and Methods

### Materials

The RALA peptide was supplied in the acetate salt form, >95% purity (Biomatik, UK, 3327.98 Da). A plasmid encoding eGFP (pEGFP-N1, Clontech) was formulated with the RALA (R-GFP). Risedronate sodium (MW:283.112 g/mol) (Sigma-Aldrich, USA) and AlexaFlour647-risedronate (AF647-RIS) (MW: 1198 g/mol) (BioVinc, California, USA) was reconstituted in TE buffer to give a final concentration of 1 μg/μL.

### Cell Lines

MDA-MB-231 and MCF-7 cell lines (ATCC, Manassas, VA) were maintained in Dulbecco’s modified eagle’s medium (Invitrogen, Life Technologies, UK) supplemented with 10% fetal bovine serum (FBS) (Invitrogen, Life technologies, UK). 4T1 (ATCC, Manassas, VA) was maintained in RPMI (Invitrogen, Life Technologies, UK) supplemented with 10% FBS. MDA-MB-231/Luc/GFP, a kind gift from Professor Ingunn Holen of University of Sheffield,^39,40^ and MG63 osteosarcoma cells (ATCC, Manassas, VA) were grown in EMEM+ (Gibco) with 10% FBS. All cell lines were authenticated by short tandem repeat profiling carried out by the supplier and routine testing revealed these cells were Mycoplasma-free.

### Animals

6 week old female BALB/c and BALB/c SCID (severe combined immunodeficient) mice were purchased from Charles River Laboratories (UK). The experimental protocols were compliant with the UK Scientific Act of 1986 and performed under project license 2881.

## Methods

### Formulation and Characterization of R-RIS NPs

RALA particles were formulated by using various RALA/RIS molar ratios. To evaluate complexation efficiency, RALA/AF647-RIS NPs were formulated through complexation of 0.1 μg AF647-RIS across a range of nmol ratios (0.5–10) and analyzed as previously described.^35^ NP stability was measured at room temperature (RT) over a period of 6 h to 12 months. Temperature stability was measured across a range of temperatures from 4 to 40 °C at 1 °C intervals. NP stability in serum (FBS) was tested over 24 h at RT. Measurements, using the Nano ZS Zeta sizer and DTS software (Malvern Instruments, UK), were reported as a mean of hydrodynamic size, zeta potential, polydispersity index (PDI) and count rate ±SEM. Particles were also assessed using transmission electron microscopy as previously described.^35^ For long-term storage, NPs were lyophilized with the addition of 20% trehalose to a final concentration of 2.5% w/v after reconstitution and stored at RT.

### Cell Viability Assays

To assess short-term viability, cells were seeded in 96-well plates at 6 × 10^3^ cells per well and incubated overnight. Cells were pretreated with Opti-MEM (Gibco) for 2 h before drug/NP treatments were added for a 6 h incubation. Treatments were replaced with fresh media and incubated for 72 h in which viability was assessed by alamarBlue (Invitrogen, Life Technologies, UK) according to manufacturer’s instructions. Long-term viability was assessed using a Clonogenic assay in which MDA-MB-231, MCF-7 (1 × 10^3^ cells/well) and 4T1 (200 cells/well) cell lines were seeded onto 6-well culture plates and treated as previously described and incubated for 7 or 14 days. Cells were washed with PBS and fixed with 0.4% crystal violet in 70% methanol. Colonies were counted manually on ImageJ software (v1.52a) using the >50 cell-exclusion criteria, and the survival fraction (SF) calculated. Viability was also assessed using a 3D spheroid model, where 2 × 10^3^ cells were seeded in ultralow attachment clear round-bottom 96-well plates, precoated with 60 μL of 120 mg/mL Poly-HEMA. After 24 h, a final concentration of 2% Matrigel was added into each well. 48 h later, cells were treated with appropriate treatments and spheroids measured daily using a plate imager for 10 days in which cell viability was evaluated via alamarBlue assay as previously described.

### Metastasis Assays

A wound scratch assay was used to evaluate the cell migration. Cells were seeded at 2.5 and 3 × 10^5^ cells/well in a 24-well plate and incubated overnight. Cells were treated as previously described. A P1000 tip was used to introduce a scratch down the center of the well, and the medium was then changed. Plates were incubated and scratches imaged on a digital microscope at relevant time points 0, 6, 24, and 48 h. All images were analyzed using ImageJ software (v1.52a) and migration of cells was expressed as a percentage of the area at *t* = 0 h. Transwell invasion and adhesion assays were performed and adapted from previous literature.^41^

### Cellular Uptake and Localization of R-RIS NPs

Internalisation of RALA/AF647-RIS NPs was confirmed by confocal laser scanning microscopy. BC cells were seeded at 1 × 10^5^ cells per slide and left to adhere overnight. Cells were treated with Opti-MEM (Invitrogen, UK) for 2 h and then treated with 0.1 μg uncomplexed AF647 or RALA-AF647-RIS. Cells were stained and analyzed as previously described.^35^

### In Vivo Studies

Female BALB/c SCID mice were implanted subcutaneously with 5 × 10^6^ MDA-MB-231/Lucif/GFP cells in 50:50 PBS: Matrigel mixture and female BALB/c mice were implanted in the fourth mammary fat pad with 5 × 10^4^ 4T1 cells stably transfected with luciferase expression plasmid (Addgene #17473). Tumour volume calculated using the following formula: *V* = 4/3π*r*^3^ [where *r* is 1/2 geometric mean diameter (GMD)] and GMD = ^3^√ (length x breadth × height). When tumor volumes reached 150 mm^3^ (between 2 and 3 weeks), mice were randomized into groups with 6 (231) or 8 (4T1) mice per group. Ten μ of designated drug (RIS or R-RIS) was given in 100 μL solution via intravenous injection. Treatment was repeated every week for 3 total injections. Mice were culled once GMD reached 15. To evaluate biodistribution, an independent experiment was performed using RALA/AF647-RIS in mice with MDA-MB-231/Lucif/GFP tumors. The left hind leg and other organs were collected 1 h after the first injection and imaged and fluorescence was recorded (*n* = 3 per group). To evaluate 4T1 distant metastasis, lungs were collected and grown in the presence of 6-thioguanine which allows for the culture of any 4T1 cells that have metastasised to the tissue as previously described^42^ Lungs were also processed and stained for H&E in the Pathology and Diagnostics lab in the Royal Veterinary Collage, kindly carried out by Ms Sue Rodway.

### Statistical Analysis

Unless otherwise stated, three technical replicates within three biological replicates were carried out for each analysis and results are presented as mean ± SEM, *n* = 3. Statistical analysis was performed using GraphPad Prism version 8.4.2. (GraphPad Software, California). Where possible, data was tested for normal distribution, but otherwise data was assumed to be nonparametric and associated tests applied. Statistically significant differences were calculated using either a Mann–Whitney unpaired two-tailed test or a Kruskal–Wallis test, with a *p*-value of ≤0.05 considered significant. Sum of square F test was performed to test the goodness-of-fit of two models in cell viability plots and conclude two populations are different (reject the null hypothesis). Where significance is not indicated, data can be assumed to have no significant differences, unless otherwise stated.

## Results

### Characterization of R-RIS NPs

**Figure 1:**
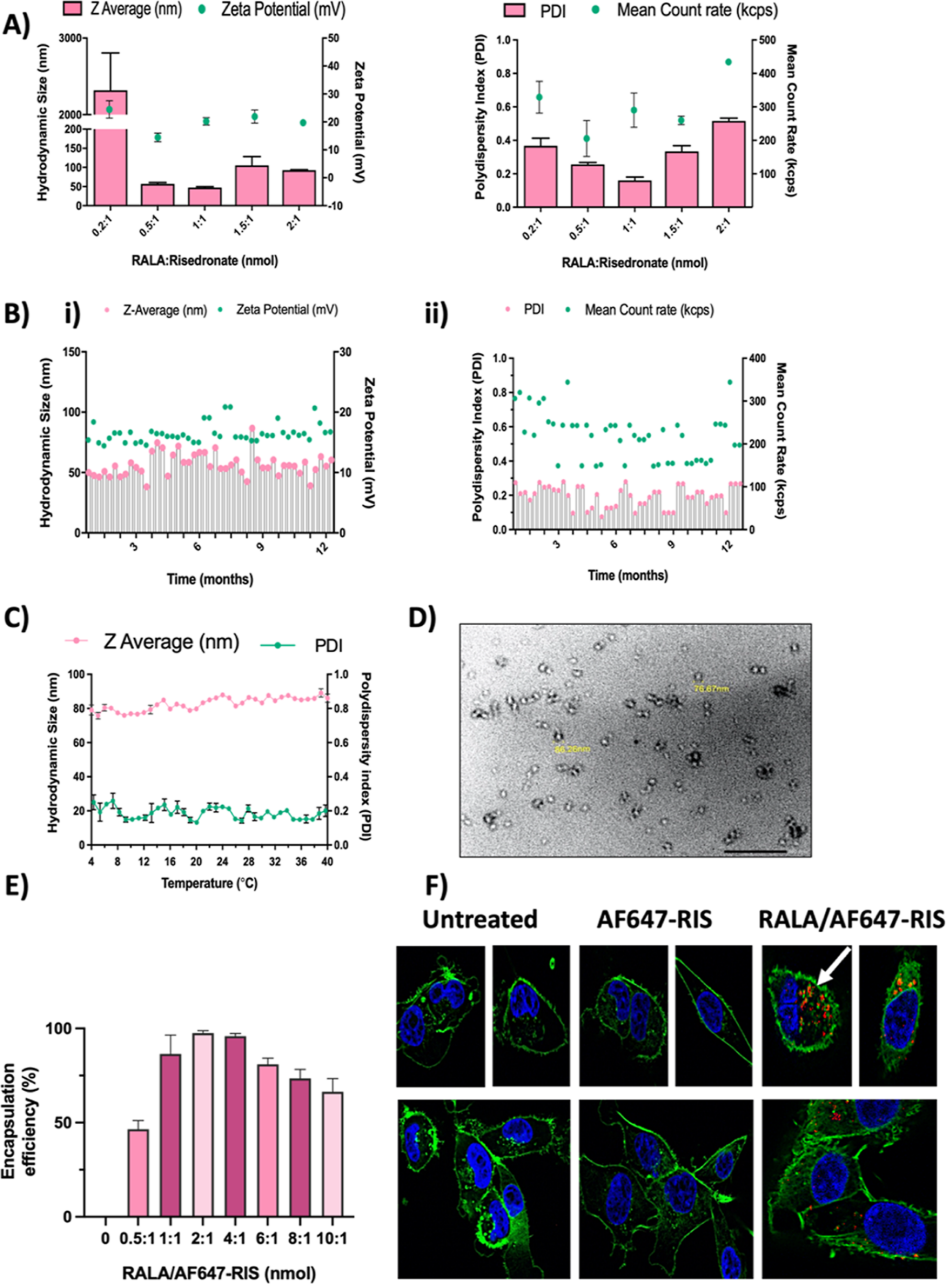
R-RIS Nanoparticle formation and characterization (A) 1 nmol of RIS was complexed with increasing nmolar ratios of RALA peptide (0.2–2) and the hydrodynamic size, mean count rate, zeta potential and PDI were evaluated using a Zetasizer. A fixed molar ratio of 1:1 RALA/RIS (60 μM) was used for further characterization studies. (B,i) Zeta potential and hydrodynamic size, mean count rate and polydispersity index (PDI) of 1:1 ratio particles was measured over 12 months. (C) Size and PDI was measured at various temperatures. (D) Representative TEM image of 1:1 ratio particles. (E) Fluorescent (red) AF647-RIS was used to evaluate complexation and cellular uptake. Particles were formulated with 0.1 μg pf AF647-RIS at increasing nmolar ratios. Complexation was calculated using AF647-RIS only as a control with 100% fluorescence and 0% complexation. (F) MDA-MB-231 cells were either untreated or treated with either 0.1 μg uncomplexed AF647-RIS or RALA/AF647-RIS nanoparticle for 2 h. Slides were fixed, stained with phalloidin (green), and mounted with DAPI (blue) mounting medium. Slides were imaged using confocal microscopy. Arrow shows internalization of AF647-RIS (red). Scale bar = 49.1 μm. All results are displayed ± SEM, *n* = 3.

A range of 0.2:1 to 2:1 nmol ratios of RALA/RIS were tested and characterized (Figure 1A). While 0.2:1 ratio was not able to form nanoparticles, all other ratios were able to form particles with a hydrodynamic diameter ranging from 50 to 150 nm with 1:1 nmol ratio being the smallest. A 1:1 ratio was determined to have the optimal NP characteristics including a hydrodynamic size <100 nm, zeta potential between 10 and 30 mV, polydispersity index (PDI) < 0.2 and a mean count rate of 100–500 kcps and stable over a year period (Figure 1B,i and S1). For storage and distribution purposes, it is necessary to lyophilize the particles. After lyophilization and resuspension, the particles remained stable and retained its optimal characteristics (Figure 1B,ii). Furthermore, the particles were stable at a range of temperatures, also important for storage and distribution (Figure 1C). The size was verified using TEM where particles formulated at a 1:1 ratio were all below 100 nm (Figure 1D). Previous studies with RALA have demonstrated that serum protein and enzymes do not decomplex RALA nanoparticles nor degrade the cargo.^34^ This stability is further supported by therapeutic responses when RALA nanoparticles are delivered systemically (intravenously) in in vivo studies.^43^ FBS was added to RALA-RIS particles for 24 h, in which the Z-average remained unchanged showing their stability in serum (Figure S2A). In order to demonstrate the release of RIS from RALA nanoparticles, AF647-RIS was used to allow for the quantification of drug complexation and release. Following serum incubation, Protease K was used to disrupt RALA particles^34^ and fluorescence values were read. As expected, when AF-647-RIS is complexed with RALA into nanoparticles, the fluorescence decreases compared to the free drug. Please note that, as RALA complexes with the cargo to condense to nanoparticles rather than encapsulates as with liposome-based nanoparticles, for example, this is not 100%. Following incubation with Proteinase K, fluorescence levels increase almost to the same level as the free drug indicating that most of the cargo has been released from the particles (Figure S2B).

### Complexation Efficiency and Cellular Uptake of R-RIS NPs

Since RALA forms a nanoparticle via a self-assembling mechanism with the cargo, encapsulation itself is not the best terminology, therefore we have changed the wording to “complexation” efficiency (CE) to hopefully help clarify this unique formulation.^34^ To evaluate complexation efficiency similar to encapsulation efficiency but more applicable to the self-assembly mechanism of RALA nanoparticles, a fluorescently labeled RIS analogue, AF647-RIS, was utilized. Drug complexation/encapsulation is important for manufacturing processes such as upscaling and to reduce the waste of expensive cargo. While the 1:1 ratio had slightly lower CE of 93% compared to the 2:1 ratio with the highest CE of 97.5%, the other ratios had a larger particle size (Figure 1E). For N-BPs to carry out their mechanism of action of inhibition of FPP synthase, they must reach the cytoplasm of the cell. Therefore, AF647-RIS was also used to determine the cellular uptake and cellular localization of the RALA NPs. We demonstrated that when MDA-MB-231 cells were treated with AF647-RIS, without RALA, negligible cytoplasmic or nuclear localization was exhibited, indicating low cell uptake. In contrast, intracellular accumulation within the cytosol was observed when treated with RALA complexed AF647-RIS (Figure 1F). Based on the characterization, complexation efficiency, and cellular uptake, a 1:1 ratio was used for the remainder of the studies.

### RALA Delivery Decreases RIS Dosage Needed to Decrease Cell Proliferation via Inhibition of Key Cell Survival Pathways

After verifying the R-RIS particles are successfully taken into the cell and accumulate in the cytoplasm, the desired location, we then investigated the cytotoxicity in two different human BC cell lines (MDA-MB-231 and MCF-7) as well as the murine triple negative breast cancer cell line, 4T1. Before investigating the cytotoxicity of R-RIS, it was important to confirm the RALA peptide has minimal cytotoxicity itself verifying pre-existing results.^34^ Since RALA cannot form NPs alone, a plasmid encoding enhanced green fluorescent protein, eGFP, (pEGFP-N1) was formulated with RALA (R-GFP) to best represent an inert/nontoxic cargo. Based on ISO 10993-5 standards, if the cell viability after treatment is less than 40% the drug has a strong cytotoxicity, while 40–60% viability is moderate and 60–80% is considered weak.^44^ Following treatments with R-GFP, MDA-MB-231 and 4T1 cell lines had a maximum viability of 89 and 86% respectively (Figure S3), indicating that the RALA carrier has limited cytotoxicity. We demonstrated that RALA peptide has minimal cytotoxic effect; therefore, any cytotoxicity coming from treatment with R-RIS will be directly from RIS alone.

**Figure 2:**
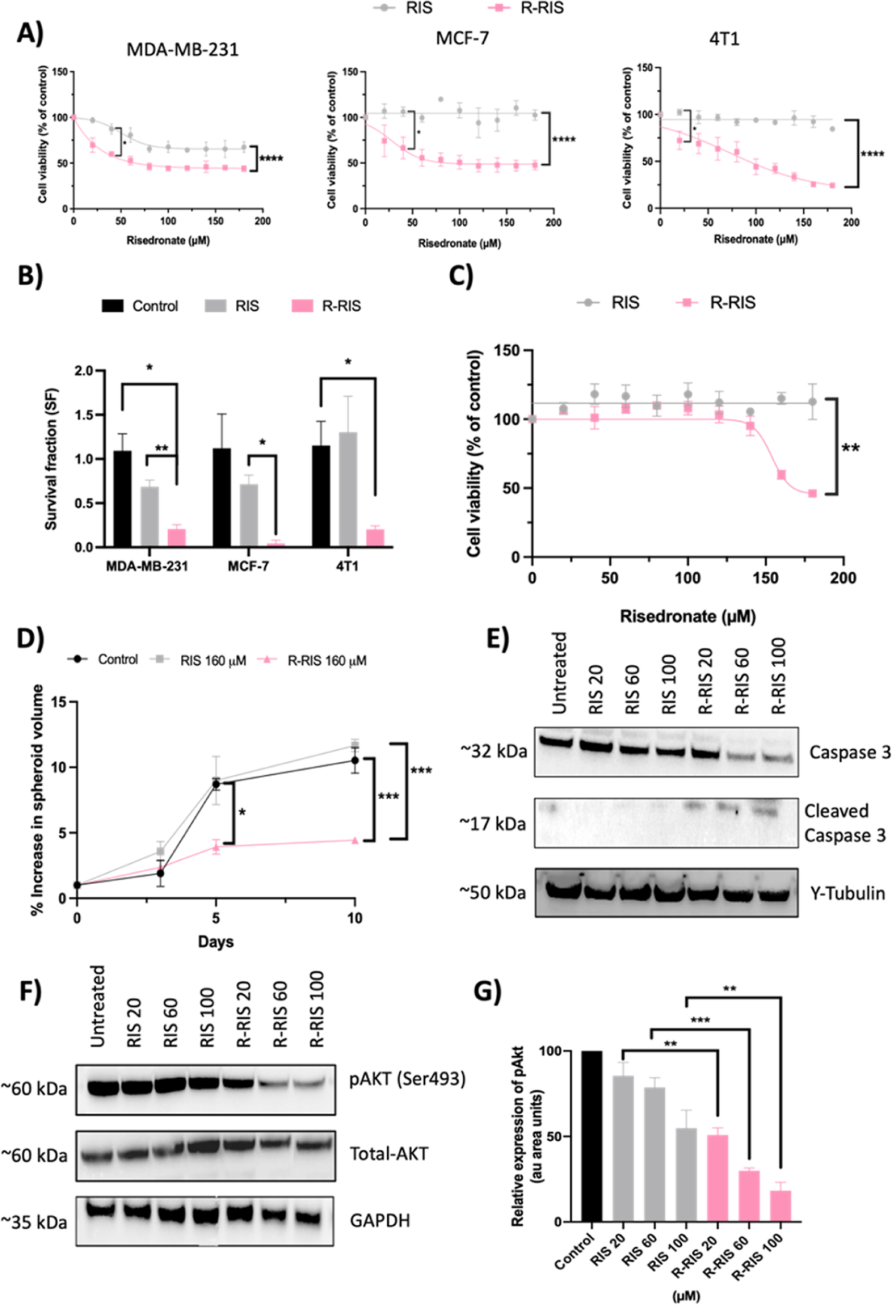
R-RIS in vitro cytotoxicity. (A) MDA-MB-231, MCF7 and 4T1 BC cell lines were treated with 1:1 nmolar ratio RIS/R-RIS particles for 6 h before replacement with complete media. Viability was assessed 72 h later via alamarBlue. Mann–Whitney unpaired two tailed test, sum of square *F* test was applied. (B) Cells were treated with 20 μM RIS/R-RIS for 6 h and then seeded in 6 well plates and left for 7 (4T1) or 14 days (MDA-MB-231, MCF-7) and stained with crystal violet. The SF was determined. A Kruskal–Wallis nonparametric test was applied. (C) MDA-MB-231 cells were seeded at 2 × 10^3^ cells/well in a 96 well round-bottom plate pre-coated with 6 μL of 120 mg/mL Poly-Hema. Matrigel was added 24 h later in a final concentration of 2.5%. Spheroids were treated with 20–180 μM and left for 10 days. Spheroid viability was measured via the Alamar Assay. A Mann–Whitney two tailed test was applied. (D) Spheroids were treated on day 4 with 160 μM RIS/R-RIS and the volume was measured over 10 days. A Kruskal–Wallis nonparametric test was applied. (E) MDA-MB-231 cells were treated with a range of RIS and R-RIS concentrations for 6 h, and protein was collected 48 h later. Protein was probed for caspase-3, cleaved caspase-3, and γ-tubulin as a loading control. (F) Cells were treated for 6 h and protein was collected. Protein was probed for pAkt, total Akt and GAPDH as a loading control. (G) Densitometry measurements were performed on expression of pAkt relative to control and normalized to total Akt. All results are displayed as ± SEM, *n* = 3. A Kruskal–Wallis nonparametric test was applied.

To evaluate R-RIS cytotoxicity in comparison to RIS alone, first a short-term assay (72 h) was performed using the three different BC cell lines. It has been shown that higher doses of N-BPs are needed to kill BC cells, with ZOL typically having one of the lowest IC_50_ concentrations (10–50 μM)^45−47^ while one study showed 20 μM of RIS was necessary to have any cell kill on MCF-7 cells with an IC_50_ of 250 μM.^48^ Therefore, a range of concentrations from 20 to 180 μM was explored in all three cell line models (Figure 2A). In MCF-7 and 4T1 cells, RIS had no cytotoxic effect, while in MDA-MB-231, RIS was able to reduce cell viability by over 25%. Interestingly, in 4T1, R-RIS became effective at 20 μM and the IC_50_ was 107.1 μM (Figure 2B), however, in both human BC cells lines (MDA-MB-231 and MCF-7), R-RIS became more effective at reducing cell viability than RIS at 20 μM, and significantly more effective at 40 μM, with efficacy plateauing at approximately 50% cell viability reduction (IC_50_ > 180 μM, Figure 2B). The inability to reduce cell kill over 50% could be due to the length of the assay, or that cells are not able to repopulate but remain viable. To see if this was the case, we performed a clonogenic assay with 20 μM RIS or R-RIS (Figure 2B and S4). In MDA-MB-231 cells, R-RIS reduced the SF by 80, 40% more than RIS alone. Similarly, in MCF-7, R-RIS reduced the SF by >90, 60% more than RIS alone. Lastly, in 4T1 R-RIS reduced the SF again over 80%, while RIS did not reduce the SF. These results demonstrate the need for a longer-term assay to show the maximum effectiveness of R-RIS on decreasing tumor cell viability.

To better model the complexity of a tumor and further explore the advantages of the RALA delivery system, the cytotoxicity of RIS and R-RIS was measured in a 3D, MDA-MB-231 spheroid model. Spheroids were treated with 0–180 μM RIS or R-RIS and viability was measured using alamarBlue (Figure S5). While RIS had no effect on spheroid viability, R-RIS significantly reduced the viability with a maximum reduction of 66% at 180 μM with an IC_50_ value of 167.9 μM (Figure 2C). From this result, spheroids were treated with an approximate IC_50_ value (160 μM). RIS showed no decrease in spheroid volume, while R-RIS significantly reduced the spheroid volume starting at day 5 and continuing through day 10 (Figure 2D).

Through the inhibition of the mevalonate pathway, N-BPs induce apoptosis via pro-caspase-3 (34 kD) activation, which in turn is cleaved into two fragments (20 and 11 kD).^49^ Cleaved caspase 3 was not detected for RIS treated MDA-MB-231 cells, while there was a dose responsive increase in cleaved caspase-3 when treated with R-RIS (Figure 2E).

Akt is a serine/threonine protein kinase that upon phosphorylation at serine 473 and threonine 308 promotes cell survival and proliferation. pAkt overexpression has been correlated with worse OS and disease-free survival (DFS) in BC patients.^50^ Furthermore, patients with pAkt overexpression have a 50% higher chance of death and 30% higher chance of recurrence compared to non-overexpressing patients.^50^ BPs inhibitory effect on the mevalonate pathway has led to studies showing a decrease in Akt phosphorylation, correlating with increased cell death.^51,52^ While 100 μM RIS was needed to see a significant reduction of pAkt, a similar reduction in expression (∼50%) was observed at 20 μM with R-RIS. When the concentration was increased to 60 and 100 μM, a ∼70 and ∼80% reduction was seen, respectively (Figure 2F,G).

### R-RIS Inhibits BC Metastatic Potential

As previously highlighted, currently clinical oncology use of BPs are restricted treatment of cancer induced bone disease, with the main mechanism of action being osteoclast apoptosis but also have been shown to reduce signals and decrease osteoclast activation induced by cancer cells.^53−55^ However, preclinical studies have also shown N-BPs can have a direct antimetastatic effect on cancer cells as well as the anti-proliferative effects already investigated.^16−21^ Therefore, we aimed to use R-RIS in early stage BC to prevent the formation of metastasis to all sites and thus investigated its effectiveness in preventing each key stage of metastasis: cell migration, invasion, and adhesion.^56^

**Figure 3:**
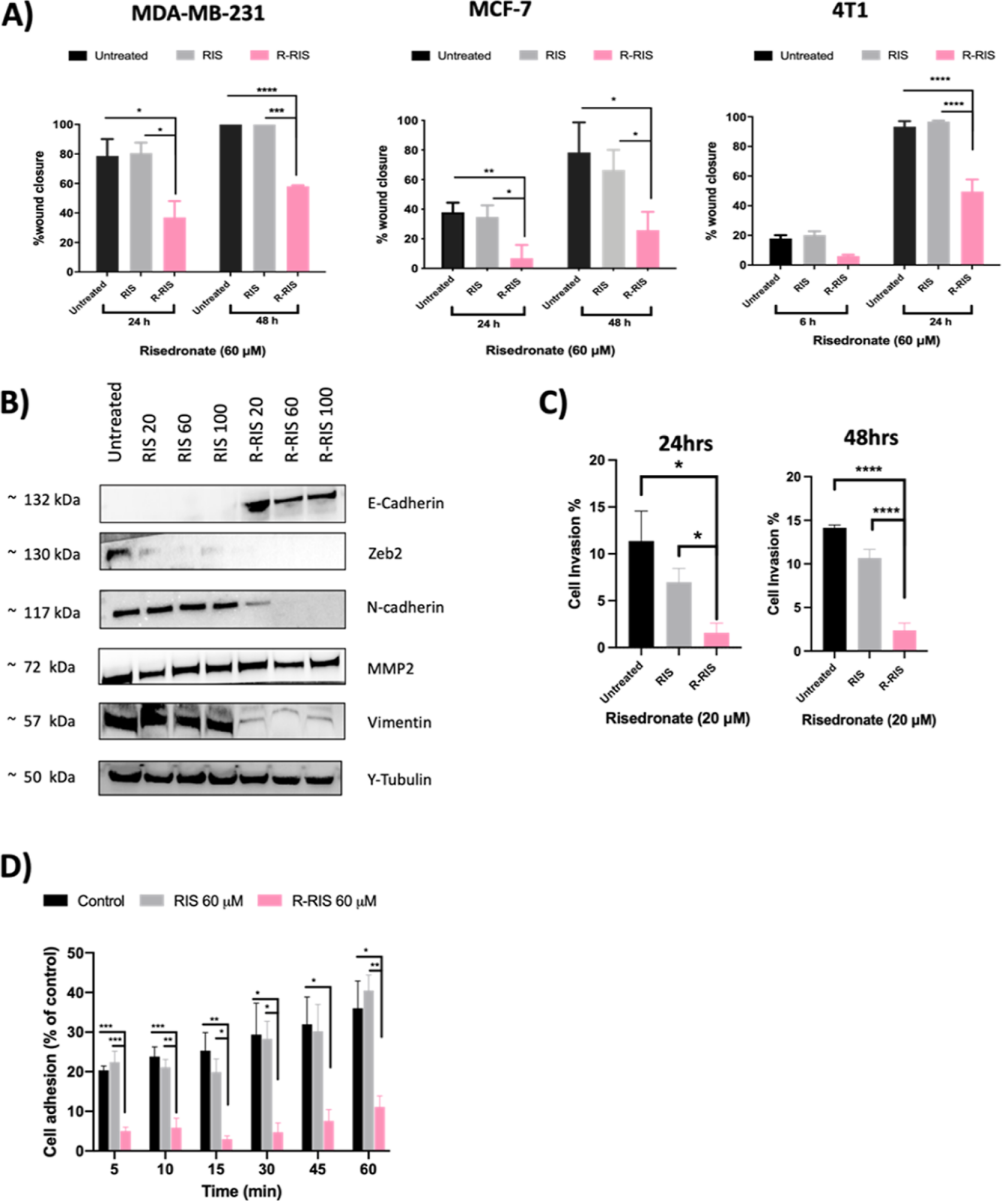
R-RIS ability to inhibit metastasis. (A) MDA-MB-231, MCF-5 and 4T1 cells were treated in a 24-well plate with 60 μM RIS/R-RIS for 6 h. A scratch was made using a p1000. Photos were taken at 24 and 48 h for MDA-MB-231 and MCF-7 cells and 6 and 24 h for 4T1 cells. The percent closure was measured. (B) MDA-MB-231 cells were treated for 6 h with 20–100 μM RIS/R-RIS and protein collected 48 h later. E-cadherin, Zeb2, N-cadherin, MMP2 and Vimentin were probed with γ-tubulin used as a loading control. (C) MDA-MB-231 cells were treated for 6 h with 20 μM RIS/R-RIS, spun down, and resuspended in serum free medium to ensure no FBS was present. 2 × 10^5^ cells were seeded in a Transwell chamber with 150 μL present in each well. Wells were stained at 24 and 48 h, and cell invasion was measured. (D) MG63 cells were seeded at 3 × 10^5^ cells/well in a 24-well plate. EGFP-transfected MDA-MB-231 cells were treated with 20 μM RIS/R-RIS for 6 h and seeded at 2 × 10^5^ cells/well on top of the MG63 monolayer. Cells were left for 1 h to adhere. Cells were washed, and fluorescence was read between 5 and 60 min to calculate the percentage of cell adherence. All results are displayed ± SEM, *n* = 3. A Kruskal–Wallis nonparametric test was applied in (A,B) and (D).

The first stage of BC metastasis is migration, which was evaluated using the wound scratch assay. Compared with untreated cells, RIS treated cells had no effect on wound closure. On the other hand, R-RIS significantly reduced wound closure compared to control in MDA-MB-231, MCF-7 and 4T1 cells (Figure 3A). Epithelial–Mesenchymal transition is critical for cells to migrate and spread, which includes cadherin switching, shown by an increase in N-cadherin followed by a decrease of E-cadherin.^57^ E-cadherin has been shown to be a key factor in cell adhesion and loss of expression has been correlated with an increase in metastasis and worse prognosis.^58^ Unlike when treated with RIS, treatment with R-RIS leads to the increase in E-cadherin expression, while reducing N-cadherin expression in a dose dependent manner (Figure 3B). ZEB2 is a key regulator of E-cadherin expression, and in cancer metastasis can down regulate E-cadherin.^59^ R-RIS was able to reduce the ZEB2 expression more than RIS. ZEBs are also a regulator of Vimentin, a mesenchymal marker associated with an increase in cell migration and invasion.^60^ The reduction of E-cadherin expression has been shown to be correlated with an increase in Vimentin.^60^ Unlike RIS, R-RIS was able to reduce Vimentin expression (Figure 3B).

The next key step of BC metastasis is invasion of the cancer cells into the surrounding tissue. Matrigel has been historically used to represent the ECM of a tumor microenvironment.^61^ Unlike RIS, R-RIS was able to reduce MDA-MB-231 invasion through Matrigel (Figure S6). R-RIS reduced cell invasion at 24 h from an average of 11.4 to 1.6% and at 48 h from 14.1 to 2.4% (Figure 3C).

Lastly, circulating cells must be able to adhere to the target metastatic site or tissue. To model this, the adherence of GFP labeled MDA-MB-231 cells, pretreated with RIS or R-RIS, to osteosarcoma cell line MG63 was assessed (Figure 3D). There was no change in the ability of MDA-MB-231 cells to adhere to MG63 osteosarcoma cells upon treatment with RIS. However, upon treatment with R-RIS there was a significant reduction in adherence (22% average reduction) compared to that of untreated and RIS treated cells.

### RALA Delivery Increases Accumulation of AF647-RIS in an MDA-MB-231 BC Xenograft Mouse Model and Significantly Decreases Tumor Growth

**Figure 4:**
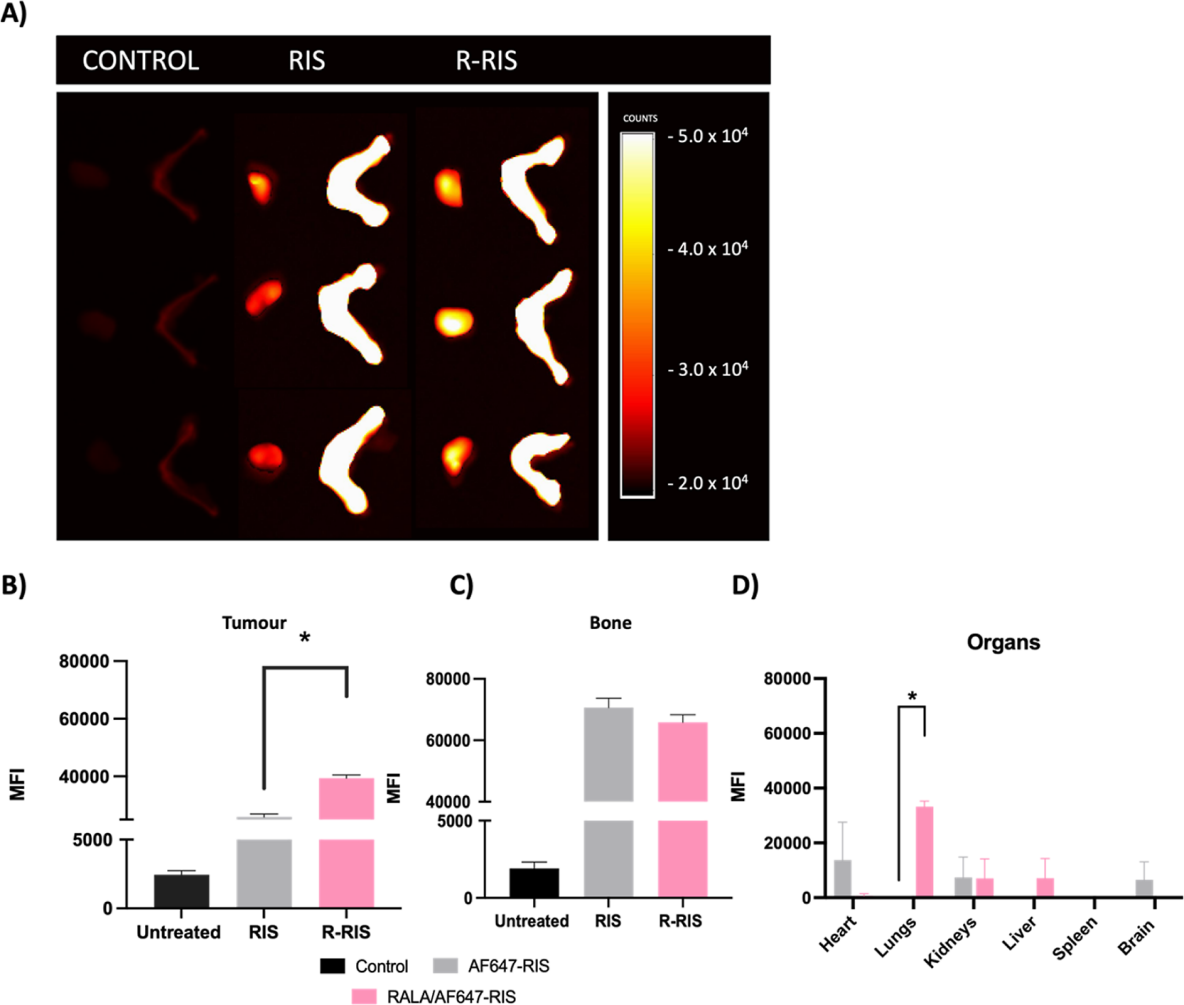
R-RIS accumulation in organs. AF647-RIS was used to assess accumulation in mouse tumor and bone. BALB/c SCID mice were implanted subcutaneously with 5 × 10^6^ MDA-MB-231 cells and grown to 150 mm^3^. Mice were treated intravenously with 10 μg of AF6476-RIS or RALA/AF647-RIS. Tumours and femurs were harvested after 1 h. (A) Fluorescent images of tumors and femurs. The relative mean drug fluorescence intensity of (B) tumors and (C) bone normalized to the weight of tumors and bone in untreated mice. (D) Organs. Results are displayed as ± SEM, *n* = 3. A Kruskal–Wallis nonparametric test was applied.

Having shown R-RIS had greater cytotoxicity and antimetastatic efficacy in vitro, we then used an in vivo model to investigate tumor accumulation of R-RIS vs RIS as this is a significant limiting factor when translating the direct antitumor effects of BPs (Figure 4A). When an equivalent dosage of AF647-RIS was delivered with RALA (10 μg), there was a significant increase in mean fluorescence intensity (MFI), or accumulation, in the tumor (Figure 4B). Along with this increase in tumor accumulation, RALA/AF647-RIS maintained similar bone accumulation as AF647-RIS (Figure 4C). To further assess the RALA/AF647-RIS accumulation, the MFI in other organs was measured (Figure 4D). RALA/AF647-RIS had slightly greater accumulation in the lungs and liver, while it showed a reduction in accumulation in the heart and brain. By binding to serum proteins attached to the surface of the particles, macrophages in the reticuloendothelial system, which 90% are in the liver, can clear NPs explaining the accumulation in the liver.^62^ Furthermore, the lungs are highly vascularized tissue and has been shown to be a place of NP accumulation in past RALA literature.^34^ While the lungs were not the intended site of accumulation, the lungs are one of the highest places of BC metastasis,^63^ which R-RIS was able to reduce. The benefit of lung accumulation could further be studied to better understand if the reduction of lung metastases is from the reduction in the primary tumor and/or if R-RIS is having a direct effect on the lung niche. Past studies investigated the effect on tissues upon RALA accumulation using RALA-P_4_-SedU_2_, a dinucleoside pro-drug that is activated in a cancer cells dependent manner and so should be inert in other tissues, thus best representing a RALA only (negative) control as RALA cannot form NPs without cargo.^43^ After treatment with RALA-P_4_-SedU_2,_ there were no signs of necrotic tissue within the gross structures of the liver, spleen, brain, lung, heart, and kidneys. Therefore, RALA is shown to not be harmful to vital organs upon its circulation and R-RIS is accumulating mostly in sites of clearance and metastasis.

**Figure 5:**
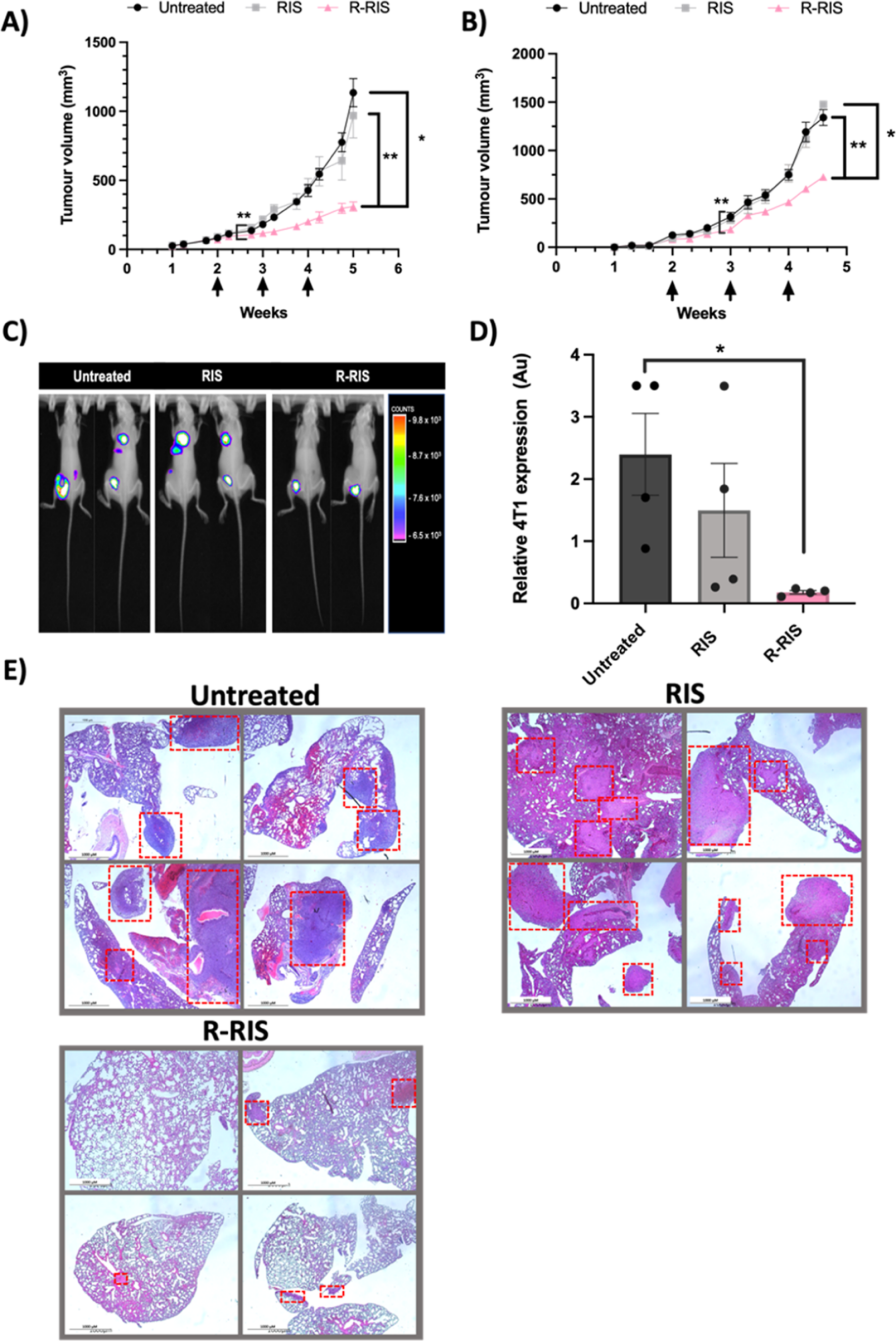
R-RIS in vivo cytotoxicity. (A) BALB/c SCID mice were implanted subcutaneously with 5 × 10^6^ MDA-MB-231 cells and grown to 150 mm^3^. Mice (*n* = 6 per group) were treated weekly intravenously with 10 μg AF6476-RIS or RALA/AF647-RIS. Tumour volume was measured. Arrows indicate when treatment occurred (week 2, 3, and 4). (B) BALB/c mice were implanted with 5 × 10^4^ 4T1 cells in the 4th mammary fat pad and tumors grew to 150 mm^3^ before treatment. Mice (*n* = 8 per group) were treated with 10 μg RIS or R-RIS intravenously weekly. Tumour volume was measured. Arrows indicate when mice were treated (week 2, 3, and 4). (C) Representative luminescent images showing 4T1 tumor and metastasis to the lungs. (D) Lungs were harvested and incubated in the presence of 6-thioguanine. After 7 days, resistant 4T1 cells were stained with crystal violet (Figure S8) and absorbance was measured 4 lungs per group were analyzed. (E) H&E staining was performed on 4T1 tumor bearing untreated, RIS treated, and R-RIS treated mouse lungs in the Pathology and Diagnostics lab in the Royal Veterinary Collage by Ms. Sue Rodway. Samples were imaged on a brightfield microscope at 60× magnification. Tumour lesions are highlighted in red boxes. Results are displayed as mean ± SEM. A Kruskal–Wallis nonparametric tests were applied.

Next, the effectiveness on the reduction of tumor volume of RIS compared to R-RIS was evaluated in an MDA-MB-231 mouse model. When treated with R-RIS, there was a significant reduction in tumor volume compared to untreated and RIS groups (Figure 5A). This is also expressed by a significant increase in doubling time and decrease in tumor weight, with no loss in body weight (Figure S7).

### Secondary Metastasis to the Lungs is Significantly Inhibited After R-RIS NP Treatment in a 4T1 Orthotopic BC Mouse Model

Given the effectiveness of R-RIS on key metastatic steps in vitro, a 4T1-luc mouse model was used to again evaluate R-RIS effectiveness on tumor burden, but also on metastasis. Like the MDA-MB-231 model, a significant reduction in tumor volume (Figure 5B) was observed when treated with R-RIS compared to untreated and RIS treatment groups. Luminescence was used to visualize 4T1-luc lung metastasis in 4 random mice in each group. A reduction in lung metastasis was observed when treated with R-RIS where no visible metastasis was seen, while 1 of 4 RIS treated and 2 of 4 untreated mice demonstrated clear metastasis (Figure 5C). These results were verified by growing lung tissue in the presence of 6-thioguanine allowing the survival and growth of 4T1 cells alone.^64^ While there was no visible metastasis in some of the mice using luminescence, colonies still formed demonstrating there was in fact metastasis although the numbers did vary indicating heterogeneity in metastatic burden which is consistent with the literature.^65^ This lower metastatic burden in the lung may not readily detectable by bioluminescence (Figure S8). Colonies were reabsorbed and quantified, showing a significant reduction in 4T1 growth from lung tissue in the R-RIS treated mice (Figure 5D). Reduction in lung metastasis was again verified by H&E where metastatic legions can be visualized in all untreated and RIS treated mice lungs, while lungs of R-RIS mice show minimal tumor growth (Figure 5E).

## Discussion

In this study, we investigated the use of RALA peptide delivery system for the delivery of risedronate to utilize BPs direct antitumor efficacy while preventing metastasis.

R-RIS nanoparticles were successfully formed having a size < 100 nm and were shown to be able to be stored long-term in variable temperatures without losing particle integrity. Furthermore, R-RIS particles had a complexation efficiency of over 90% while liposomal N-BPs have 1–6% and 66% in self-assembling PEGylated particles.^66,67^ We showed that R-RIS was able to increase the cytoplasmic localization of RIS into BC cells.

Once we successfully formulated R-RIS particles, we then investigated its effectiveness in inhibiting cancer cell proliferation and metastasis. We showed that unlike RIS, R-RIS was effective in decreasing BC cell proliferation, migration, invasion, and adhesion at the doses assessed. The concentrations needed to decrease cell viability and metastasis were similar or lower than more potent ZOL.^45−47^ While many studies only showed decreased metastasis in vivo, we were able to reduce lung metastasis and decrease primary tumor volume.^28,68,69^

We first investigated the cytotoxicity of R-RIS in vitro in three BC cell lines (MDA-MB-231, MCF-7, and 4T1). Literature has shown that all N-BPs need a high dosage to have an impact on cell viability. Studies have shown N-BPs have an IC_50_ ranging from 35 to 2000 μM^46^ and even the most potent N-BP, Zoledronate had an IC_50_ of 10–50 μM in vitro.^45−47^ One study showed that 1 μM of RIS only reduced cell proliferation by up to 20%.^47^ In our studies, R-RIS was able to reduce cell viability by up to 80% at a concentration of 20 μM, comparable to ZOL.^45−47^ We further demonstrated R-RIS effectiveness in a 3D model, comparable with previous results with RALA-Alendronate.^35^

It has been shown that BPs induce apoptosis via a caspase-dependent pathway shown by an increase in caspase-3 activation.^46,49,68,70^ We evaluated the RIS effectiveness of inducing apoptosis via the activation and subsequent cleavage of caspase-3. One study showed that MCF-7 treatment with 1 μM of 4 different N-BPs (clodronate, pamidronate, ibandronate, and zoledronate) increased caspase activity.^70^ Unlike with RIS, treatment with R-RIS led to a dose dependent increase in cleaved caspase-3 expression. Furthermore, R-RIS was as, if not more effective, in reducing pAkt expression at a similar concentration than previous studies with ZOL.^51^

R-RIS potential to inhibit metastasis in vitro was investigated. The first stage of metastasis is the migration of the cells. RIS treated cells had no effect on wound closure, while R-RIS significantly reduced wound closure compared to the control in all 3 lines. This is one of the first times that the use of BPs for the inhibition of migration has been investigated. For cell migration to occur, the cancer cells must alter their cell–cell and cell-ECM adhesion properties. One study showed that when MDA-MB-231 cells were treated with ZOL, mesenchymal marker N-cadherin expression decreased while epithelial marker E-cadherin expression increased.^19^ We similarly showed that R-RIS was able to increase E-cadherin and decrease N-cadherin. The next key step of BC metastasis is invasion of BC into the surrounding tissue. One study showed that 1 μM Zoledronate achieved 90% inhibition while NE-10244 (active pyridinium analogue of risedronate) achieved 60%. In another study, 1 μM of ZOL only achieved 50% inhibition with a maximum inhibition of ∼80% using 100 μM.^21^ In our study, 60 μM, RIS achieved <40% invasion inhibition, while R-RIS achieved over 80%. By using R-RIS, we achieved a similar inhibition of invasion as the more potent Zoledronate, while at a lower dosage. Lastly, migrating cells must be able adhere to the target metastatic site or tissue. Literature has shown that N-BPs can inhibit MDA-MB-231 cell adhesion to bone matrix in a dose dependent manner (1–100 μM).^16−18^ RIS alone did not inhibit MDA-MB-231 ability to adhere to bone, while R-RIS significantly reduced MDA-MB-231 adherence, which is consistent with previous studies which also showed that when bone cells were pretreated there was no reduction in cancer cell adherence.^18^ This confirms that BPs can have a direct effect on BC cells in the reduction of metastasis. This emphasizes the necessity of investigating the accumulation of BPs in the tumor as well as the bone to gain full benefit from BP therapy, as we have shown R-RIS was able to do in vivo.

There have been multiple studies investigating the use of BPs in BC mouse models,^71^ however, most have focused solely on their effect on preventing bone metastasis through modulating osteoclasts. Since 1995, multiple BC mouse studies have shown the efficacy of various BPs in reducing bone metastasis and osteolytic lesions. In 1995, in a nude mouse model where MDA-MB-231 cells were injected intracardially which forms osteolytic bone metastasis, RIS was first demonstrated to be effective in reducing bone metastasis.^9^ Since then, Hiraga and team have conducted many studies showing the ability of BPs to reduce bone, lungs and liver metastasis in MDA-MB-231^69^ and 4T1 models.^68^ Although success was found in the metastatic setting, even ZOL, the most potent BP, showed no benefit on the primary tumor in both MDA-MB-231^69^ and 4T1 models.^28^ Similarly, we showed RIS was ineffective in reducing primary tumor growth in both an MDA-MB-231 and 4T1 model. Meanwhile, we demonstrated the increased tumor accumulation of R-RIS reducing tumor volume in both an MDA-MB-231 and 4T1 model. This increased tumor accumulation can be explained, in part, by the enhanced permeability and retention (EPR) effect, whereby NPs via passive targeting accumulate more readily in tumors due to their defective lymphatic drainage and leaky vasculature.^72^ This can be supported from past work, showing RALA complexed P_4_-SedU_2_, a dinucleoside, RALA-P_4_-SedU_2_ had a lower plasma *C*_max_ than the drug alone. Therefore, it can be inferred that RALA was being taken up into and retained in the organs more readily than free drug.^43^ Furthermore, R-RIS was able to reduce lung metastasis. We showed using AF647-RIS, that when delivered with RALA, there was an increase in tumor accumulation as well as maintaining equal bone accumulation. Therefore, we have shown that by using the RALA delivery system, we have increased tumor accumulation of RIS and thus able to reduce primary tumor burden and prevent metastasis to other sites of metastasis while maintaining its bone targeting properties.

## Conclusions

BC impacts one in every eight women worldwide, and metastasis is a significant driver of unsuccessful treatment that results in patient death. Bisphosphates (BPs) have been used for the treatment of bone metastasis, yet their direct antitumor properties have not been fully utilized due to poor bioavailability. Through this study, we have shown that we are able to produce effective R-RIS nanoparticles that were optimal in size while having a higher complexation/encapsulation over other published nanoformulations. The formulation of R-RIS particles is simple, inexpensive, and easily scalable, with a low loss of material. We showed that R-RIS nanoparticles had far superior anticancer effects than RIS both in vitro and in vivo. R-RIS was able to have the same accumulation in the bones as free RIS while having significantly more accumulation in the tumor itself. Unlike RIS, R-RIS was able to inhibit tumor growth as well as metastasis. Furthermore, by unlocking RIS direct antitumor effects, the treatment is no longer limited by the lack of bone turnover found in premenopausal women. Furthermore, using RALA, we observed similar metastasis effects and better direct tumor effects than the more commonly used ZOL, at a lower dosage, which ultimately will reduce potential toxicity in patients. R-RIS is a good candidate for the treatment of primary early BC and the prevention of metastasis in pre- and post-menopausal women in combination with standard of care chemotherapy.

## Supporting Information Available

Nanoparticle characterization; nanoparticle stability; nanoparticle toxicity; clonogenic assay; 3D cell culture assay; cell invasion assay; in vivo data; and ex vivo metastasis data (PDF)
