# Supplementary material for: Development and Characterization of a Peptide-Bisphosphonate Nanoparticle for the Treatment of Breast Cancer
Source: Mol Pharm. 2024 Aug 28;21(10):4970–82. doi: 10.1021/acs.molpharmaceut.4c00299 (PMC11462496; doi:10.1021/acs.molpharmaceut.4c00299)
Supplement: Supplementary file 1 — mp4c00299_si_001.pdf [file mp4c00299_si_001.pdf]

Development and Characterisation of a Peptide-Bisphosphonate Nanoparticle for the Treatment of Breast Cancer

Kimberley Glass, Cory Fines, Paula Coulter, Lynn Jena, Helen O. McCarthy, Niamh Buckley*

Queen's University Belfast, School of Pharmacy
97 Lisburn Road BT9 7BL Northern Ireland, United Kingdom

*Niamh Buckley,
Queen's University Belfast, School of Pharmacy
97 Lisburn Road BT9 7BL Northern Ireland, United Kingdom
n.obrien@qub.ac.uk

Supplemental Figures

			Size (d.nm):	% Intensity:	St Dev (d.nm):
Z-Average (d.nm):	120.9	Peak 1:	140.0	89.8	74.74
PdI:	0.361	Peak 2:	3531	10.2	1226
Intercept:	0.592	Peak 3:	0.000	0.0	0.000
Result quality :	Good				

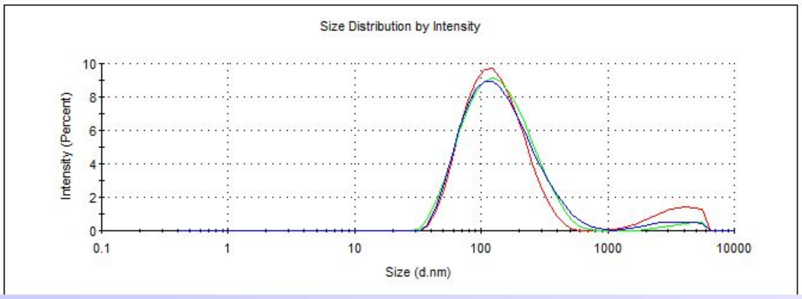

Supplemental Figure 1:

Example representation of polydispersity index spectra of RALA nanoparticles.

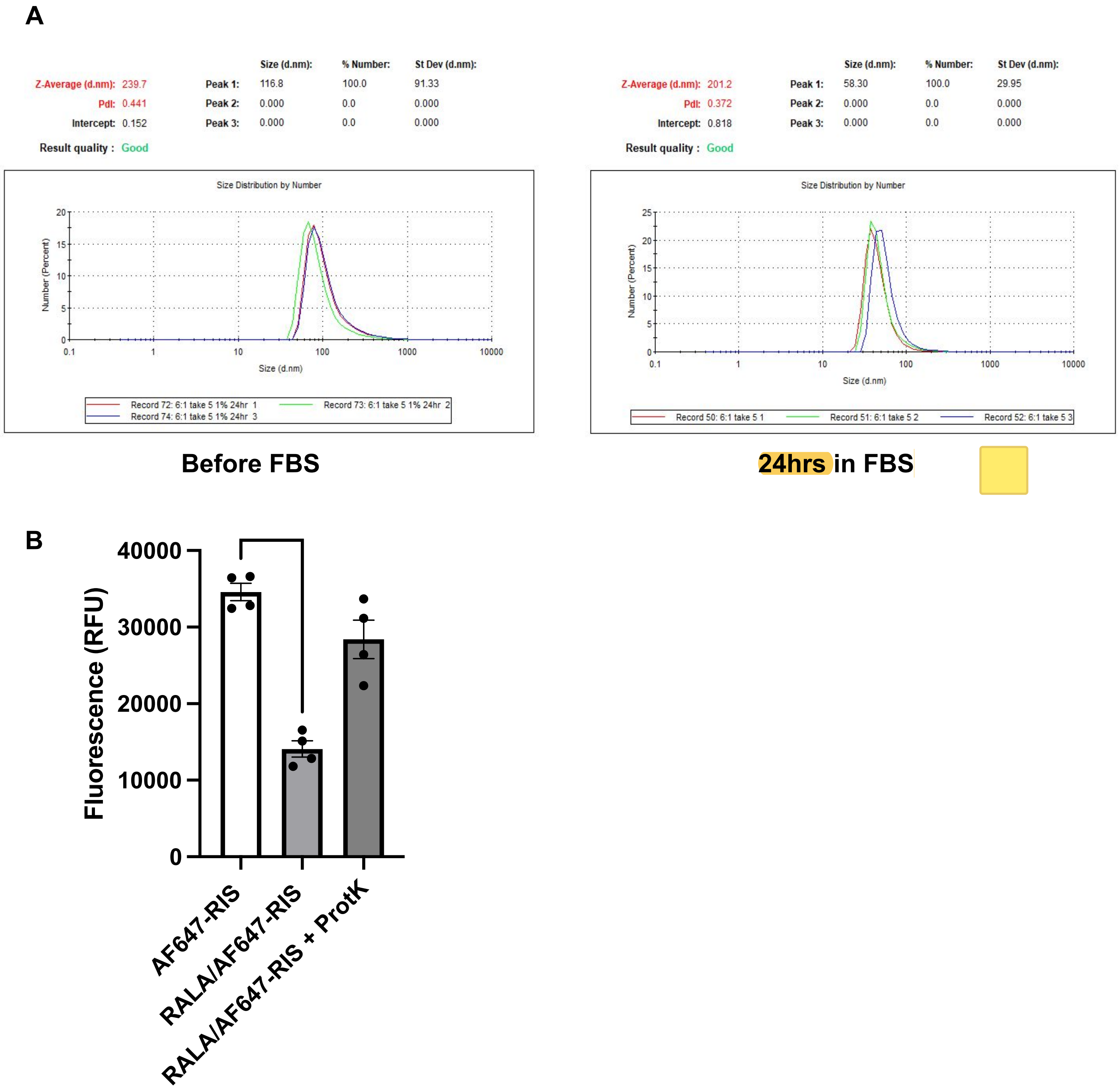

Supplemental Figure 2:

A. Representative DLS spectra of RALA-RIS nanoparticles before and after incubation with serum for 24hr.
B. Bar graph showing fluorescence at 647nm of free AF647-Ris, RALA-AF647Ris and RALA-AF647Ris following digestion with Proteinase K

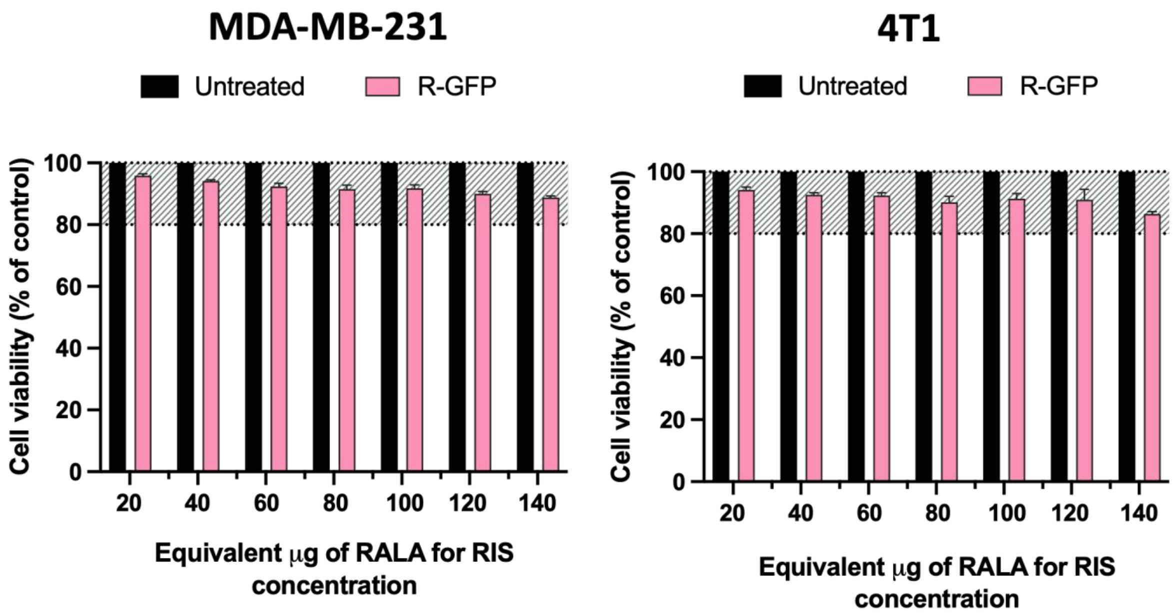

Supplemental Figure 3:

Nanoparticles were formed with increasing amounts of RALA peptide formulated with 1 ug pEGFP-N1 at NP:10. MDA-MB-231 and 4T1 cells were treated for 6 hours before replacement with complete media. After 72hrs cells viability was measured with Alamar Blue.

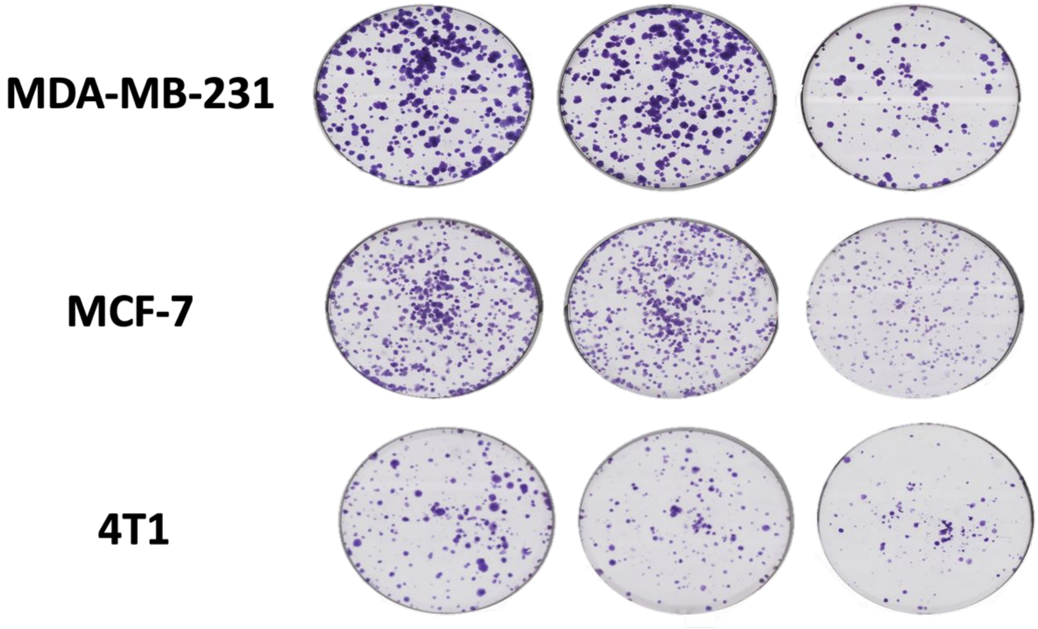

Supplementary Figure 4:

Cells were treated with 20uM RIS/R-RIS for 6hrs and then seeded in 6 well plates and left for 7 (4T1) or 14 days (MDA-MB-231, MCF-7) and stained with crystal violet. Representative images of crystal violet stained 6 well plates.

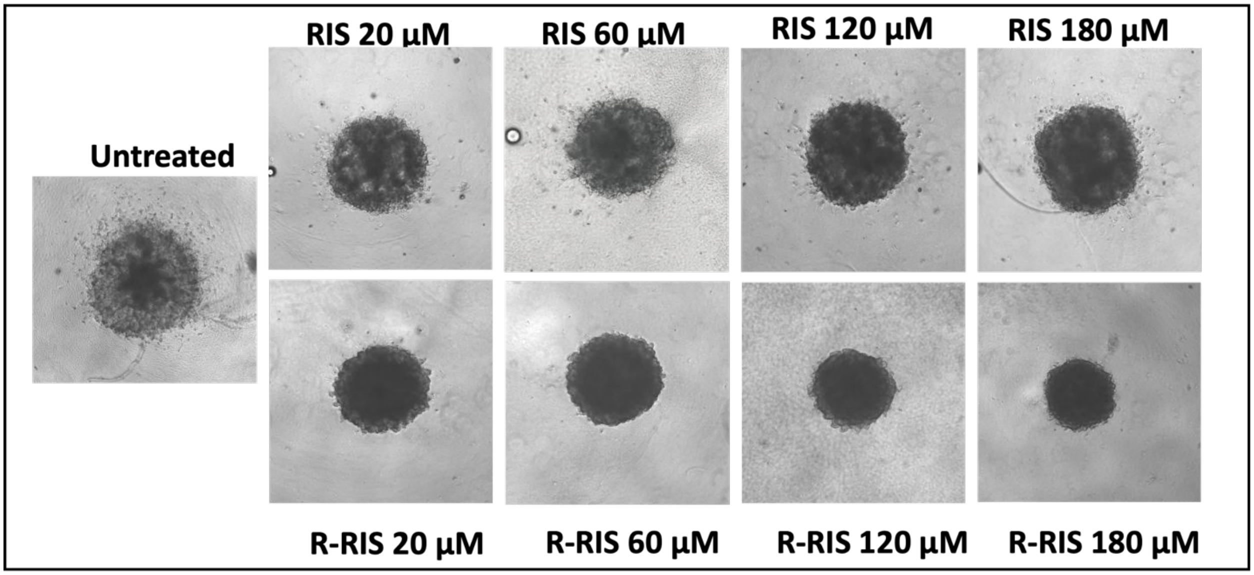

Supplementary Figure 5:

MDA-MB-231 cells were seeded at 2,000 cells/well in a 96 well round bottom plate pre-coated with 6ul of 120mg/ml Poly-Hema. Matrigel was added 24hrs later in a final concentration of 2.5%. Representative images of spheroids treated with 20-180uM of RIS/R-RIS.

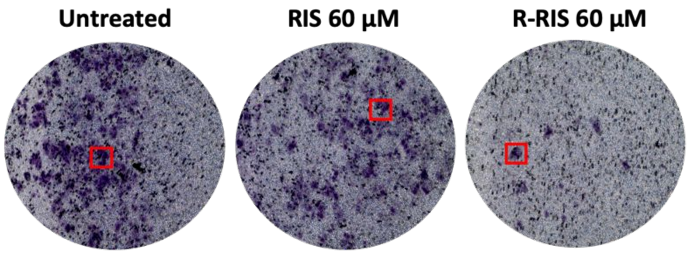

Supplementary Figure 6:

MDA-MB-231 cells were treated for 6hrs with 20 mM RIS/R-RIS, spun down, and resuspended in serum free medium to ensure no FBS was present. 250,000 cells were seeded in a Transwell chamber with 150ml present in each well. Wells were stained at 24 and 48hrs and cell invasion was measured. Visual representation of cell invasion at 48hrs is shown. Red boxes highlight stained cells that have undergone invasion through the membrane.

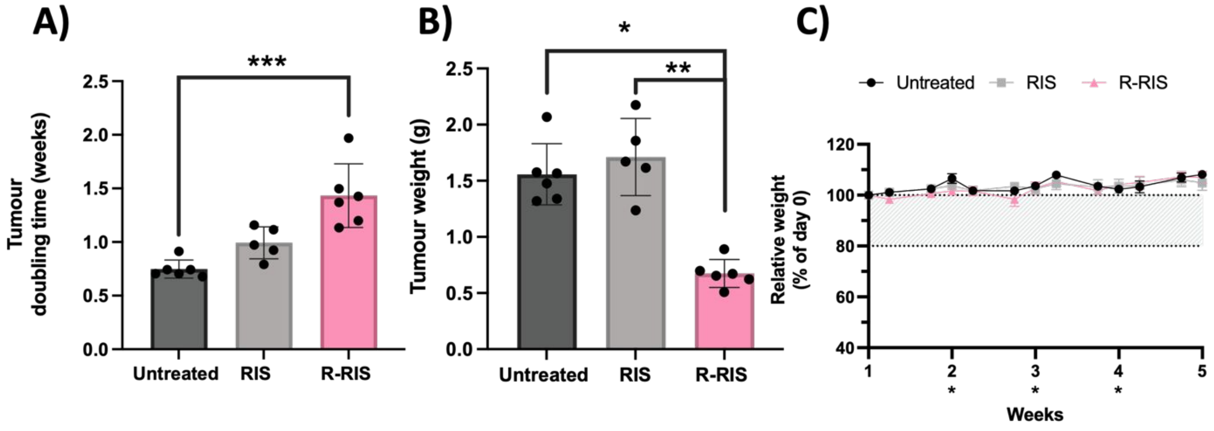

Supplementary Figure 7:

BALB/c SCID mice were implanted subcutaneously with 5x10^6 MDA-MB-231 cells and grown to 150mm^3. Mice (n=6 per group) were treated weekly intravenously with 10ug AF6476-RIS or RALA/AF647-RIS. A) Tumour Doubling time B) Tumour weight C) Body weight. All results are displayed +/- SEM. A Krustal-Wallis non-parametric test was applied.

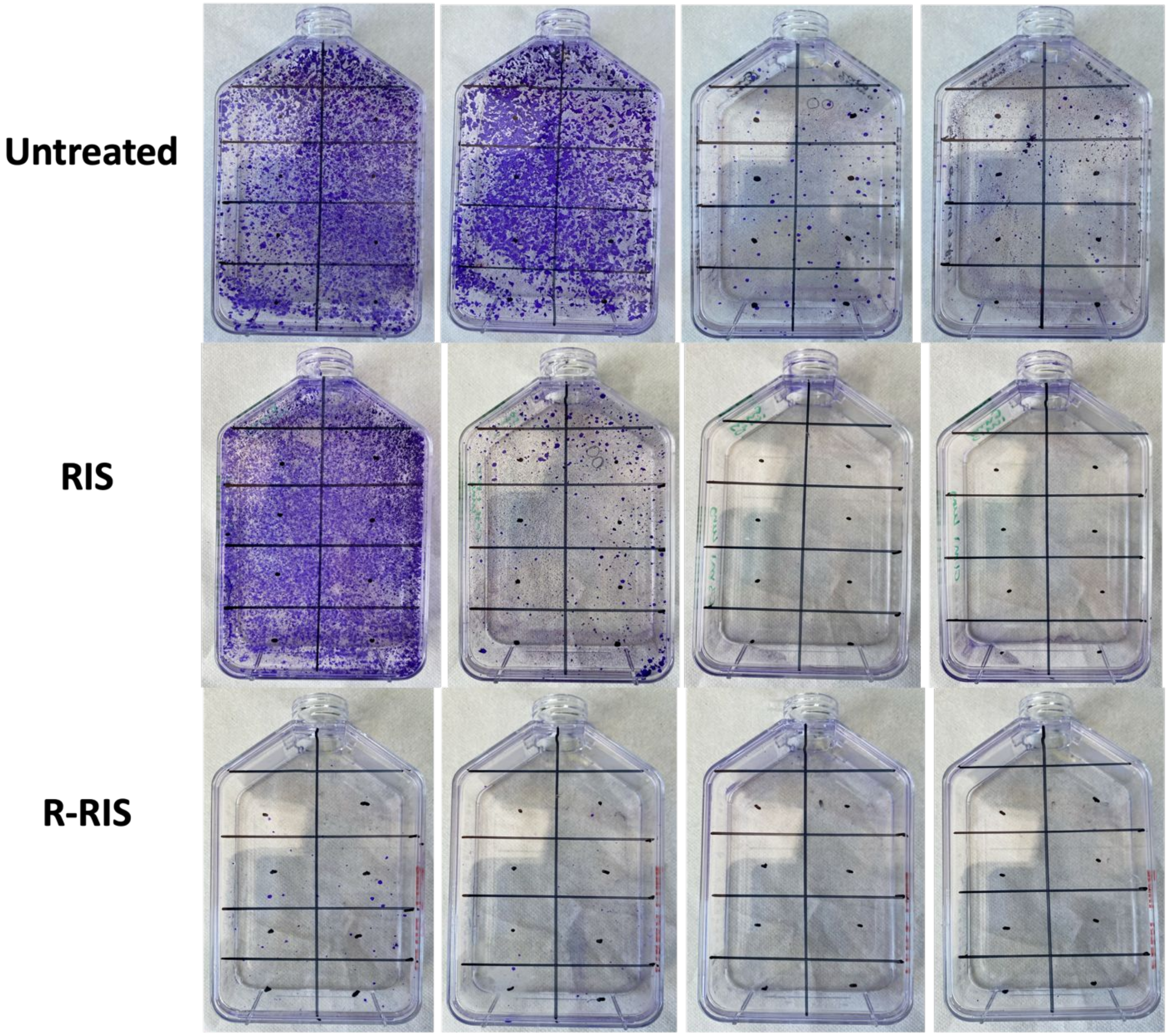

Supplementary Figure 8:

BALB/c mice were implanted with $5x10^4$ 4T1 cells in the 4th mammary fat pad and tumours grew to $150mm^3$ before treatment. Mice (n=8 per group) were treated with 10ug RIS or R-RIS intravenously weekly. Lungs were harvested and incubated in the presence of 6-thioguanine. After 7 days, resistant 4T1 cells were stained with crystal violet and images taken.
